# The Transcriptional Repressor Domain of Gli3 Is Intrinsically Disordered

**DOI:** 10.1371/journal.pone.0076972

**Published:** 2013-10-17

**Authors:** Robert Tsanev, Kalju Vanatalu, Jüri Jarvet, Risto Tanner, Kristi Laur, Piret Tiigimägi, Birthe B. Kragelund, Torben Østerlund, Priit Kogerman

**Affiliations:** 1 Department of Gene Technology, Tallinn University of Technology, Tallinn, Estonia; 2 National Institute of Chemical Physics and Biophysics, Tallinn, Estonia; 3 Structural Biology and Nuclear Magnetic Resonance Laboratory, University of Copenhagen, Copenhagen, Denmark; 4 Molecular Pharmacology, Zealand Pharma A/S, Glostrup, Denmark; University of South Florida College of Medicine, United States of America

## Abstract

The transcription factor Gli3 is acting mainly as a transcriptional repressor in the Sonic hedgehog signal transduction pathway. Gli3 contains a repressor domain in its N-terminus from residue G106 to E236. In this study we have characterized the intracellular structure of the Gli3 repressor domain using a combined bioinformatics and experimental approach. According to our findings the Gli3 repressor domain while being intrinsically disordered contains predicted anchor sites for partner interactions. The obvious interaction partners to test were Ski and DNA; however, with both of these the structure of Gli3 repressor domain remained disordered. To locate residues important for the repressor function we mutated several residues within the Gli3 repressor domain. Two of these, H141A and H157N, targeting predicted helical regions, significantly decreased transcriptional repression and thus identify important functional parts of the domain.

## Introduction

The expression of human genes is controlled by numerous transcription factors. Depending on the physiological context genes are activated by transcriptional activators or repressed by transcriptional repressors. During the development of organism, the fine-tuning of gene expression depends on an intricate balance between positive and negative regulators. There are three transcription factors (Gli1, Gli2 and Gli3) in the Sonic hedgehog signal transduction pathway [1]. In their central part these proteins contain a conserved DNA binding domain (DBD) consisting of five zinc-fingers. The structure of the DBD from Gli1 has been solved by X-ray analysis [2]. Here, the zinc-fingers 3, 4 and 5 closely contact the DNA with fingers 4 and 5 determining the target DNA sequence (GACCACCCA) [3] of Gli binding. The first finger does not contact DNA and the second finger only partially interacts with DNA. All the three Gli proteins contain a transcriptional activator domain (TAD) at their C-terminus [4], [5]. For transcriptional repression Gli proteins utilize at least two mechanisms. The first one, common to all three Gli proteins, is dependent on Sufu and histone deacetylase [6]. The second one is histone deacetylase independent, involving the domain that we previously identified and named the repressor domain (RD) [7]. Here, we investigate the structure and partner interactions of this domain. The RD is only present in the N-terminus of Gli2 and Gli3 but not in Gli1 [4], [7]. An alternative, third mechanism of negative transcriptional regulation by Gli3 has been suggested to involve Ski and histone deacetylation, indicating a general mechanism for all Gli proteins [8]. *In vivo*, Gli3 transcriptional repression has been shown in Sufu knockout mice, suggesting Sufu independent repression mechanism [9].

The proteins or protein domains involved in transcriptional regulation often belong to the class of intrinsically disordered proteins (IDP) or regions (IDR) [10], [11]. Structural disorder provides advantages in fulfilling the dynamic processes of gene regulation and signal transduction, and is more frequent in multicellular organisms, suggesting complex regulatory mechanisms [12], [13].

In some instances the IDR undergoes binding-coupled folding and becomes functional [14], whereas in other cases a larger degree of fuzziness in the complex is possible [15]. Hence, the IDPs recognise and interact with their partners by a number of mechanisms. A predominant one utilizes short sequence elements displaying higher structural propensities in the disordered, unbound state. These sequence elements are known as molecular recognition features or MoRFs [16]. It has been reported that many IDPs contain low-stability structural elements [17], [18].

In this work we have studied the biophysical properties of the RD of Gli3 as previously mapped to span residues G106 to E236 [7]. Our results indicate that it constitutes an intrinsically disordered region. In addition, we have investigated its interactions with various potential partners. Sufu is a known partner and negative regulator of all Gli proteins, but the Gli3 interaction site for Sufu (S_333_YGH_337_ [19]) does not overlap with the RD. Another partner of Gli3, the Ski protein, has also been linked to the repression function of Gli3 [8]. The interaction site for Ski has been mapped to N-terminus of Gli3 (1–397). This site potentially overlaps with the RD, thus making Ski a possible target for Gli3RD.

Some transcription factors bind DNA through their IDR [20]. Therefore, we investigated whether Gli3RD, as IDR, interacts with DNA. The transcription factor p53 has two DNA-binding domains: a disordered C-terminal domain (CTD) and a structured core domain [21]. The CTD binds to DNA in a sequence-independent manner and slides along DNA. This assists the core domain in finding its consensus site. The DNA binding of RNA polymerase II (RNA pol II) [22] is facilitated by its predominantly disordered CTD [18]. This domain is proline rich and bears some resemblance to the proline rich sequence of Gli3RD. The DNA binding of RNA pol II CTD is not sequence specific but is dependent on the intercalation of the aromatic ring of tyrosine into the DNA strands [22]. There are eight tyrosine residues in the Gli3RD sequence and some of them have the same spacing between tyrosine and proline as in the CTD of RNA pol II. This suggests that if Gli3RD is binding DNA, it might also be sequence-unspecific. It is known that Gli3 binds to its target genes through the Zn-finger domain that recognises a specific DNA sequence- the Gli consensus site. It has not been observed that Gli3 is able to bind any other sequence in addition to its known DNA binding sequence [23]. We reasoned that if the RD was able to bind DNA, it should occur in a sequence-independent manner, otherwise an additional consensus sequence for Gli3 should have been described.

The Gli3RD contains several histidines. Histidines can coordinate Zn^2+^ ions and according to Karlin [24] there are six classes of histidine ligands. Within Gli3RD, positions H121/H157 and H141/H147 resemble class II and I Zn^2+^-ligands, respectively. For that reason we decided to test whether these histidines coordinate Zn^2+^. We mutated these histidines and tested the repressor function of the resultant variants.

A useful method for studying protein folding is in-cell NMR where the spectrum is measured directly inside the living cells in physiologically relevant conditions. Measurements are often carried out by expressing the target protein in *E.coli*. We may suppose that the *E.coli* intracellular environment is more native-like even for an eukaryotic protein than the dilute solution conditions. It is known that the conformation of IDPs may be sensitive to molecular crowding of the environment [25], [26], [27]. Therefore it is appropriate to carry out the studies as in-cell NMR. The ^15^N,^1^H-HSQC NMR spectrum of IDP has low dispersion of signals in the ^1^H-dimension within a narrow region around 8 ppm. Certain residues give NMR signals which are easily recognizable due to their distinct positioning. These are the cross-peaks of glycines, the mirrored signal from the side chains of asparagines and glutamines and the signal from the side chain of arginines. The prolines are residues that do not give rise to a signal in the NMR spectrum.

We have described here Gli3RD as an IDR. In regard to its function as a transcriptional repressor we aimed to investigate whether Gli3RD binds Ski or DNA. In addition, we also examined the secondary structure induction of Gli3RD. In a functional assay, H141 and H157 were identified as important functional parts of the domain.

## Results and Discussion

### Gli3RD is predicted to have both order and disorder features, with a mostly disordered N-terminal part

**Figure 1:**
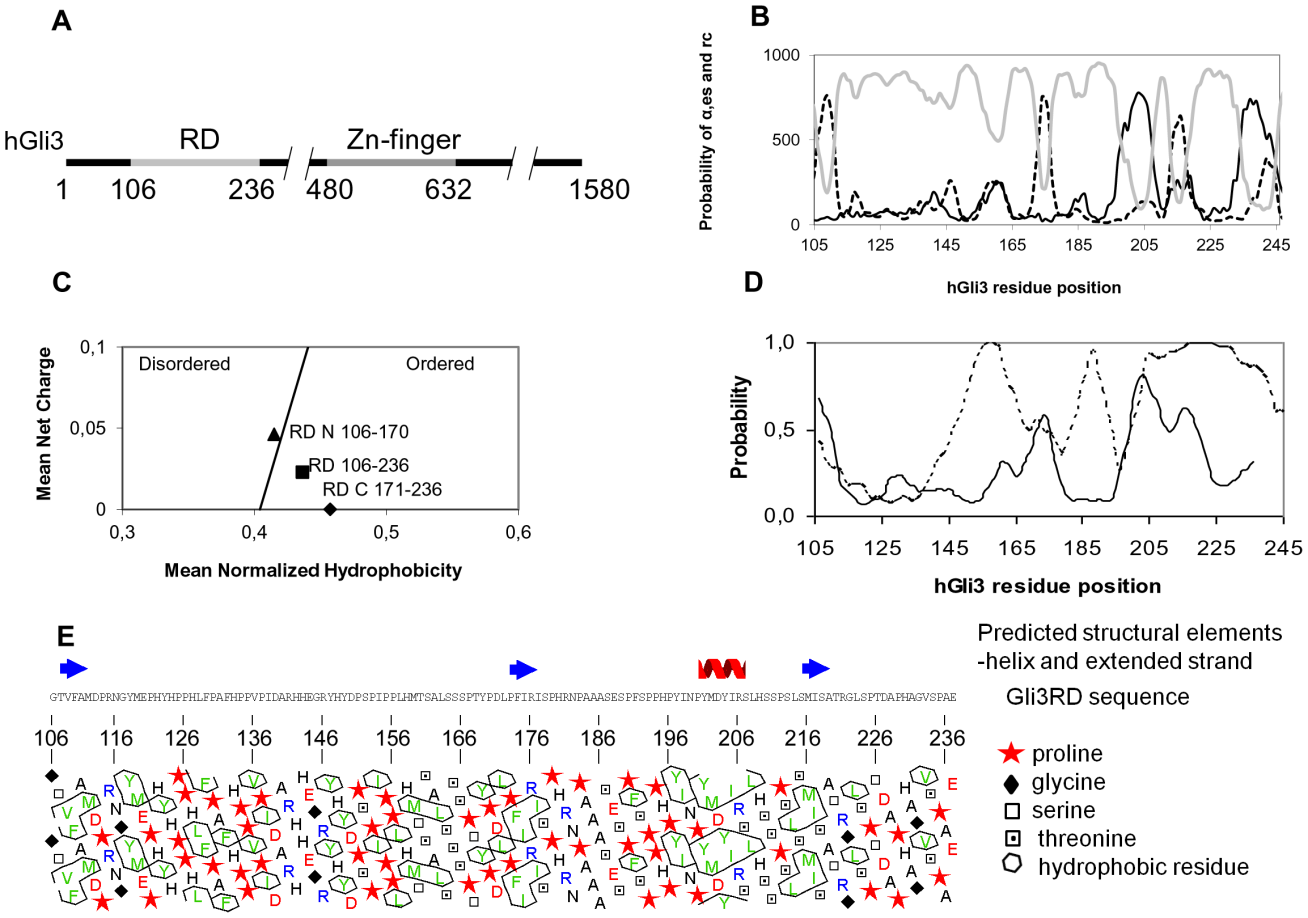
Sequence analysis of human Gli3RD. (A) The relative position of RD within Gli3 from residues G106 to E236. (B) Gli3RD structure prediction with the Hierarchical Neural Network prediction method. The probability for α-helix (α) formation is shown with (-), extended strand (—) and random coil (-). (C) Net charge/hydrophobicity plot of the Gli3RD, as well as of the N- and C-terminus shown separately. The charge/hydrophobicity diagram is divided into two regions by a line (-) corresponding to the equation (R) = 2,743(H)-1,109. Proteins on the left and right side of the diagram are predicted to be disordered and ordered, respectively. (D) Prediction of disorder by PONDR (VL XT algorithm) is shown with a dashed line (—) and the binding regions prediction by ANCHOR is indicated with a line (-). (E) The hydrophobisity cluster analysis combined with secondary structure prediction and amino acid sequence of the human Gli3 repressor domain.

The transcription factor Gli3 contains an RD in its N-terminus that represses gene expression. We previously identified the Gli3RD to reside between residues G106 to E236 [7] as depicted in Fig. 1A. Sequence analyses of Gli3RD revealed higher than average content of histidines, serines and prolines, particularly in the N-terminal region from residues G106 to D170. This makes the sequence being potentially capable of binding to Zn^2+^ ions, post-translationally modified, and extended and rigid. The Gli3RD sequence appears to be mostly disordered when analysed by disorder-predicting algorithm (VL XT, PONDR, [28]), especially its C-terminal region (Fig. 1D, dashed line), while the N-terminal part appears more ordered. The order-predicting programme, Hierarchical Neural Network, HNN method [29], (Fig. 1B, grey line) also reveals Gli3RD to be predominantly disordered, containing several sequence stretches with higher order probability, mostly within the C-terminal part of Gli3RD. These potentially ordered elements are two short extended strands from positions F173 to I176 and S214 to S217 (Fig. 1B, dashed line) and one short α-helix at position N198 to L207 (Fig. 1B, black line). One potentially extended strand stretching from residue G106 to M111 (Fig. 1B, dashed line) was predicted in the N-terminus of RD. The loss of probability of disorder around position P178 (Fig. 1D, dashed line) coincides with the predicted extended strand at positions F173 to I176 (Fig. 1B, dashed line). The next minimum of disorder probability at position I197 (Fig. 1D, dashed line) corresponds to the predicted α-helix at position N198 to L207 (Fig. 1B, black line). Using ANCHOR programme [30] to predict protein binding sites prone to undergo disorder-to-order transitions, we identified two sites in the C-terminal region from residue D170 to I174 and residues P199-T219 (Fig. 1D, black line). The first site (D170-I174) is close to the predicted extended strand (F173-I176) while the second site (P199-T219) overlaps with the sequence prone to form an α-helix (N198-L207), (Fig. 1B and 1E). These sites can probably undergo structuralisation upon binding to a functional partner, while remaining disordered in a free state. When analysed by the method of Uversky [31] that assesses intrinsic disorder based on the ratio between mean hydrophobicity and mean net charge, Gli3RD as a whole was predicted to be an ordered domain. Accordingly, the hydrophobicity/charge balance does not support strongly disordered regions, as the N-terminal part (residues106–170) lies at the order/disorder boundary and C-terminal part (residues171–236) is situated at the order site (Fig. 1C). The hydrophobisity cluster analysis (HCA) performed with the metaserver MeDor [32], (Fig. 1E) reveals the absence of hydrophobic core which, combined with the high proline content will contribute to the extended state of Gli3RD. The hydrophobic residues within RD colocalise with proline residues to form proline- and hydrophobic residues- rich sites connected by short sequences depleted from these residues. While the proline rich sites are rigid, the linkers seem to be more flexible. Based on the HCA, (Fig. 1E) such linker sequences are D138-R145 and T159-S165. One hydrophobic cluster is seen around position 200, corresponding to the predicted α-helix (Fig. 1E).

It has been found that most of the proteins involved in transcriptional regulation are either completely disordered or contain large regions of intrinsic disorder [20]. Consistent with this, Gli3 also contains extensive regions of intrinsic disorder. The whole N-terminus up to the Zn-fingers (residues 1-480), including RD (residues 106-236), shows high probability for disorder (PONDR, HNN; data not shown). Taken together, we conclude that the RD contains properties of both an ordered and disordered protein, suggesting that folding-upon-binding may occur by utilizing the two C-terminal anchor sites.

### Gli3RD is intrinsically disordered in a native-like environment as determined by in-cell NMR

**Figure 2:**
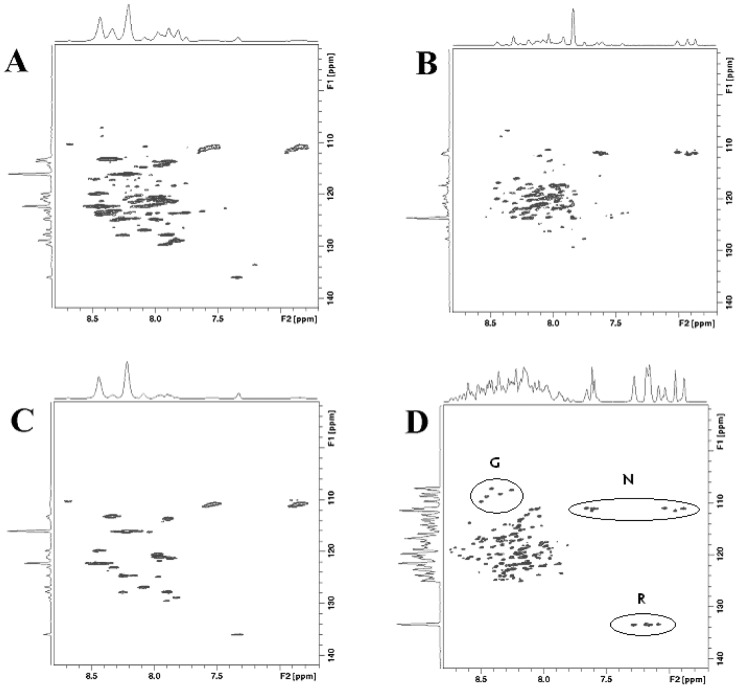
The ^1^H-^15^N HSQC NMR spectra. (A) in-cell spectrum of *E.coli* expressing Gli3RD, (B) Gli3RD in solution at pH 7.4, (C) in-cell spectrum of uninduced *E.coli* and (D) Gli3RD in solution at pH 5.8. The cross-peaks from glycines (G), the mirrored cross-peaks from the side chains of asparagines (N) and the signals from the side chain of arginines (R) are denoted in circles.

To determine the structure of Gli3RD, this 15.575-kDa protein domain was His-tagged and expressed in *E. coli*. The NMR spectrum of Gli3RD expressing cells (Fig. 2A) is qualitatively similar to that of the purified protein, measured at pH 7.4 (Fig. 2B). All of the proton peaks lie within a narrow window of 8 ppm that is typical for unfolded peptides. The *E. coli* control cells, not expressing Gli3RD, are shown in Fig. 2C. At physiological pH we detected half of the Gli3RD cross peaks (Fig. 2A) as opposed to the spectrum of the purified protein at pH 5.8 (Fig. 2D). It is possible that a folded fraction bound to a protein partner may have too broad peaks to be seen in the NMR spectrum, or that the missing peaks are not seen due to broadening caused by intra-molecular interaction. However, since Gli3RD is expressed in a heterologous system it most probably has no endogenous binding partner but may interact with the intracellular components unspecifically [33]. The sequence of Gli3RD is proline rich, rendering its structure more rigid and extended thereby making intra-molecular interactions unlikely. Most probably the missing resonances are due to the faster proton exchange occurring at higher pH [34].

In the NMR spectrum of purified Gli3RD we could recognise all five glycines and the side chains of the three asparagines (Fig. 2D), while the signals from the side chains of the seven arginines were visible but overlapping (Fig. 2D). At physiological pH two glycines are detectable (Fig.2B), and they are also visible in the in-cell spectrum (Fig. 2A). This analysis provides supporting evidence that the structure of Gli3RD is disordered in the intracellular environment.

### Gli3RD does not bind Ski

It is known that IDRs/IDPs can undergo binding-coupled folding with their functional partners. For that reason we attempted to induce Gli3RD folding using its potential partner Ski, previously reported to interact with the N-terminal part of Gli3 (residues 1–397) that contains the repressor domain (106–236) [8]. To accomplish this, a Ski variant (residues G88 to Y291) was expressed and purified as a maltose-binding (MBP) fusion protein and then added to the ^15^N-labelled Gli3RD. The NMR spectrum of Gli3RD remained unchanged in the presence of MBP-Ski (data not shown) indicating no major structural changes of Gli3RD in the presence of Ski. We then tested their interaction by co-immunoprecipitation analysis using tagged over-expressed proteins. However, we did not detect an interaction between Gli3RD and Ski (data not shown). Ski utilizes HDACs to achieve transcriptional repression, suggesting a general mechanism of repression for all three Gli proteins. However, the identified RD does not use HDACs to achieve repression and is found in Gli2 and Gli3 only, but not in Gli1 [7]. Probably the interaction site for Ski is located more upstream from RD, possibly overlapping with the Sufu-binding site (S_333_YGH_337_). Alternatively, Ski might interact with Gli3 not directly but through Sufu.

### Gli3RD does not bind DNA

**Figure 3:**
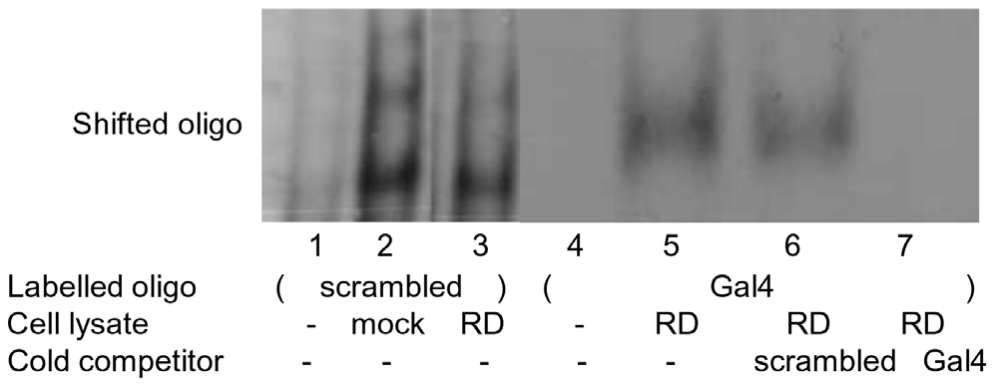
Electrophoretic mobility shift analysis of Gli3RD and DNA binding. Cell lysate from Gli3RD or mock-transfected HEK293 cells incubated with labelled scrambled sequence oligonucleotide is shown in line 3 and line 2, respectively. As a negative control, the labelled scrambled oligonucleotide was loaded alone, without a cell lysate, on line 1. Labelled Gal4 binding site (BS) oligonucleotides were used to confirm the presence of Gal4 DBD tagged Gli3RD. On line 4 the Gal4 BS oligonucleotide is loaded alone. The DNA-protein complex formed by the Gal4 BS oligonucleotide and Gal4 DBD tagged Gli3RD is indicated on line 5. The shifted complex was competed out by unlabelled Gal4 BS oligonucleotide (line 7). Scrambled sequence oligonucleotide did not compete out the complex (line 6).

It has been emphasised that structural disorder is widespread among transcription factors and nucleic acids binding proteins [10], [11]. Thus, we subsequently investigated whether GLi3RD interacts with DNA or otherwise. For this we used CD spectroscopy, because this technique has been successfully employed to study the binding of architectural proteins (HMGB1, H1) to DNA [35]. To test if DNA could induce binding-coupled folding of Gli3RD we obtained the CD spectrum of purified recombinant Gli3RD alone or together with a 21 base-pair scrambled sequence DNA oligonucleotide or plasmid DNA to exclude DNA size requirements. After addition of oligonucleotide or plasmid DNA at an stoichiometric ratio of 1∶1, no dramatic change in spectral appearance was observed, suggesting that Gli3RD had remained disordered (data not shown). However, although DNA did not induce binding-coupled structure in Gli3RD (CD may not detect low-affinity binding), these two molecules might still interact. We decided to test this possibility by Electrophoretic Mobility Shift Assay (EMSA) and expressed the Gli3RD in HEK293 cells as a Gal4DBD-tagged protein. The cell lysate of HEK293 cells (Fig. 3, lane 2) or a Gli3RD-expressing cells (Fig. 3, lane 3) was incubated with a ^32^P-labelled 21 base-pairs scrambled DNA oligomers. If Gli3RD was able to bind the labelled oligonucleotide a band corresponding to the DNA-Gli3RD complex should appear. In Fig. 3, comparison of lanes 2 and 3 (with and without Gli3RD, respectively) revealed no additional bands, implying that Gli3RD does not bind DNA. To ascertain that Gli3RD was present in the cell lysate, we used labelled Gal4BS oligonucleotides binding to the Gal4DBD tag of Gli3RD. The corresponding complex, comprising Gli3RD-Gal4DBD protein / Gal4BS oligo is seen in Fig. 3, lane 5, as a specific band. The specificity of this complex was proved by out-competing with unlabelled Gal4BS oligonucleotide (Fig. 3, lane 7) but not by scrambled oligo (Fig. 3, lane 6). We noticed that Gli3RD did not alter the DNA binding of its tag, Gal4DBD. Therefore, we exclude the mechanism where Gli3RD functions at the DNA level, by preventing the Gli3 Zn-finger to bind to DNA.

### The repression activity of Gli3RD is lost in H141A and H157N mutants

**Figure 4:**
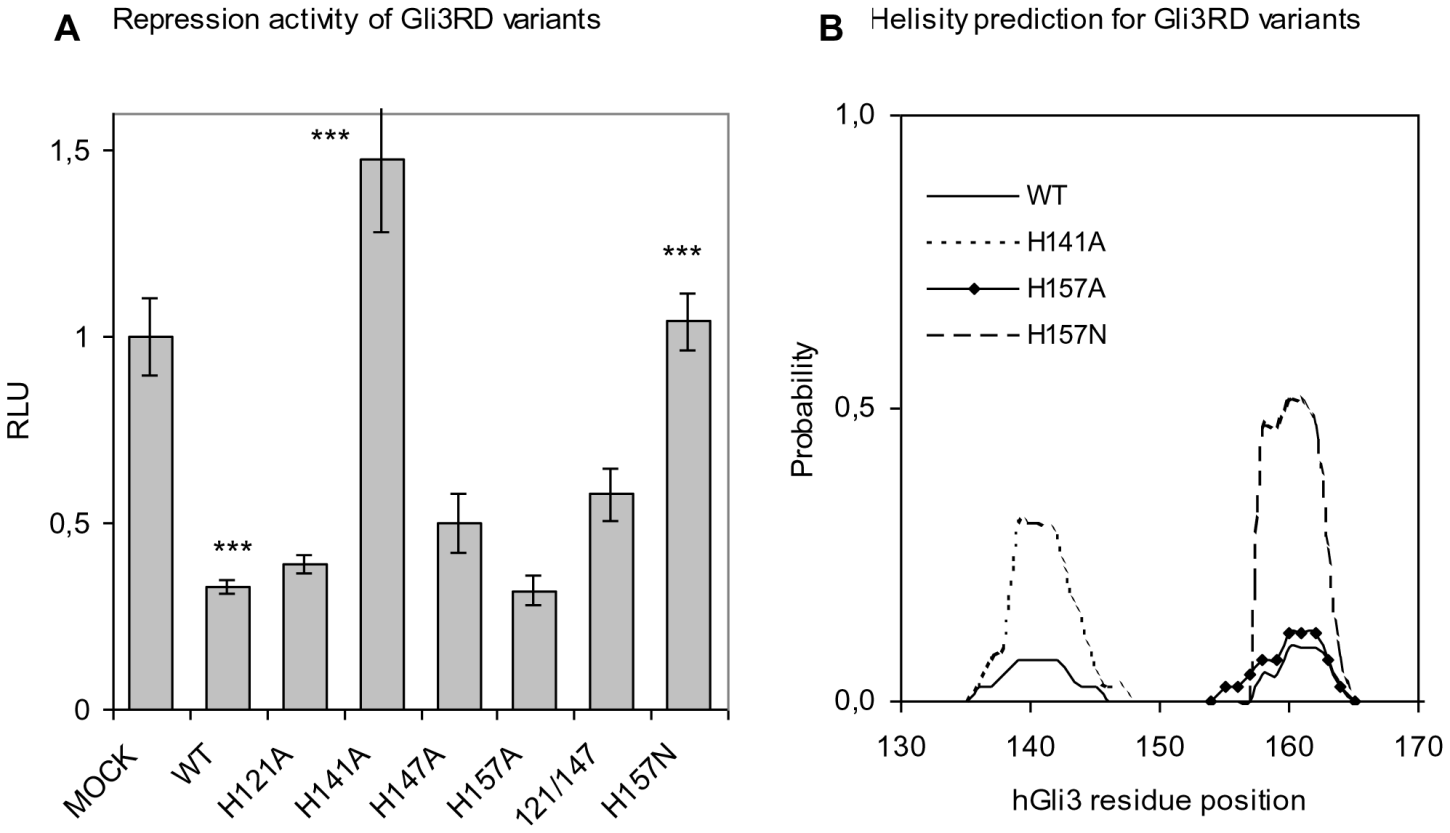
The luciferase reporter activity together with different Gli3RD variants. (A) All Gli3RD constructs were expressed as Gal4 DBD fusions in HEK293 cells and their repressor activity is compared to that of Gal4 DBD alone (mock). The activity of Gla4-Gli3RD is shown as WT. The analyses were performed three to five times and error bars show standard deviations. Statistically significant were WT vs MOCK, WT vs H141A and WT vs H157N, p<0,001, One-Way ANOVA with Dunnett's Multiple Comparisons Test. (B) The Agadir prediction of helical behaviour for Gli3RD variants.

Karlin and Zhu describe six classes of Zn-ligands [24]. The positions of H121/H157 and of H141/H147 resemble classes II and I, respectively, of Zn^2+^-binding ligands. We investigated whether the repression function of Gli3RD is dependent on Zn^2+^-binding. This was addressed in a functional assay by comparing the activities of wild type to histidine-mutated Gli3RD variants. For such mutational analysis we chose H121 and H157 since they resemble the first and the third histidines from class II site of Zn^2+^-binding ligands (H_121_xH and a third histidine_157_ distant in the sequence) [24]. We also selected H141 and H147 because they resemble the third and the second histidines from a class I site (HExxH_141_xxGxxH_147_). Moreover, the two sites are also positioned in two sequence-stretches predicted by HNN to form low-populated α-helices (and lower than the helix in the C-terminal part). We mutated H121, H141, H147 and H157 to alanines separately and in a double mutant H121/147A, where both classes of Zn^2+^-ligands were expected to be affected. In addition, the H157 was substituted for asparagines, since asparagine and histidine have similar, but not identical, space requirements and hydrogen bonding capabilities. However, asparagine is unable to coordinate Zn^2+^-ions. Asparagine was also chosen because, contrary to H157A, its substitution significantly alters the predicted local helicity at that site which might be involved in partner recognition. All proteins were expressed as tagged versions from DNA in HEK293 cells. The constructs encoded Gal4 DBD-fusion proteins and we measured their effect on transcriptional activity of Gal4 binding site-containing luciferase reporter (Fig. 4A).

The mutation of the first histidine (H121) from class II did not affect the repression function. The second histidine mutation (H147) from class I, also left the repression function unaltered. To verify that repression is Zn^2+^-independent we combined the H121 and H147 mutations in a double mutant H121/147A. In this mutant, both putative Zn^2+^-ligand classes were affected. Despite targeting both classes I and II, the repression function remained intact. The activity of the double mutant is slightly different but falls within the range of other mutants (Fig. 4A).

Upon mutating the histidines H141 or H147 that resemble the class I Zn-ligands, we observed a significant loss of repression activity in the H141A only. Thus, it is possible that H141 participates in Zn^2+^ coordination together with a more distant histidine. On the one hand, Gli3RD is a proline rich sequence, making its structure extended and therefore any long-range intramolecular interactions more difficult. On the other hand, H141 is at the peak of a low-populated predicted α-helix that reaches a minimum at H147 (Fig 4B). Since the H141A significantly changes the structural ensemble of the above α-helix and correspondingly affects the repressor function, we speculate that it might constitute a protein interaction site or functionally important region with distinct structural dynamics. In the case of H157, the activity of the domain was preserved in H157A variant whereas it was lost in H157N. By analogy with H141A, while the H157N variant increases the predicted helicity by more than 300%, the same does not hold true for the functionally intact H157A variant. Therefore we speculate that the change in the structural ensemble of the predicted α-helix, may respectively either impair or enhance partner recognition via altering a specific MoRF [16]. This remains to be proven when the Gli3RD interaction partner is known. The observed loss of activity in the H141A and H157N variants points that these residues most probably constitute important functional parts of the repressor domain.

Our analyses were performed using the RD expressed alone (fused to a 6xHis-tag) and not as part of the full-length Gli3 protein or as a larger part encompassing the RD and the surrounding region. It cannot be ruled out that in the composition of the entire protein this domain has diminished spatial freedom due to stabilizing inter-domain connections and is therefore more folded than we observed [36]. However, homologous domains are present in different proteins, meaning that the information about their function and folding is mainly coded within the domain sequence, and only to a minor extent by the surrounding context of the protein. It cannot be ruled out that, although the domain appears disordered on its own, it can undergo structural changes upon interaction with a binding partner in the context of the full-length protein only. Nevertheless, as the Gli3RD domain on its own has repressor function (Fig. 4A; [7]), the latter is not likely.

In conclusion, we have described the Gli3RD as an intrinsically disordered domain. The RD does not bind and fold in the presence of neither Ski nor DNA. The H141A and H157N mutants point to important functional parts of the repressor domain, possibly involving α-helical MoRFs. The H157 shows partial functional plasticity, tolerating its substitution for alanine but not for aspargine.

The pursuit for binding partner and complex structure will continue with this and other variants of the Gli3RD. An interesting option would be to study the repressor complex forming on the promoter region of one of the target genes of GLI proteins, i.e. *PTCH* or *GLI1*, as well as the one forming with the DBD itself.

## Materials and Methods

### Sequence analyses

Sequence analyses were carried out as described by Uversky [31]. The mean net charge of a protein was determined as the absolute value of the difference between the numbers of positively and negatively charged residues divided by the total number of residues. The mean hydrophobicity was defined as the sum of the normalised Kyte-Doolittle hydrophobicities, divided by the total number of residues. These values were then plotted together with disorder-order boundary, which was defined as (charge) = 2.743 (hydrophobicity) – 1.109. Sequences were also analysed using series neural network predictors as implemented in PONDR VL-XT (pondr.com) [28]. For the secondary structure prediction the Hierarchical Neural Network prediction method was used [29] (http://npsa-devel.ibcp.fr/NPSA/npsa_hnn.html).

### DNA constructs

For production of recombinant protein the cDNA for human Gli3RD (residues 106 to 236) was cloned into pET11C (Novagen, USA). An N-terminal 6xHis-tag was designed into the primer followed immediately by the RD sequence. The cDNA for human Ski (residues 88 to 291) was cloned into pMAL vector. For expression of Gli3RD in mammalian cells, we used the pFA Gli3RD construct described in [7] where the Gli3RD cDNA was in frame with the DBD of yeast Gal4 (Stratagene, La Jolla, CA). We used the pMN-Luc plasmid containing a thymidine kinase promoter with five tandem repeats of the yeast GAL4 binding sites that control expression of the firefly luciferase gene as a Gal4 reporter construct. The Gli3RD mutants were cloned in pFA vector produced as described in QuikChange protocol (Stratagene).

### Protein expression and purification

Expression of recombinant 6xHis-Gli3RD (Gli3RD) was carried out in *E. coli* strain BL21. For ^15^N labelling the cells were grown in minimal medium supplemented with glucose and ^15^NH_4_Cl in a volume of 1 litre in fermentor and expressed for 5 hours by induction with 0.5 mM IPTG at 30°C. Cells were harvested by centrifugation. The pellet was resuspended in 40 ml of ice-cold lysis buffer (100 mM NaH_2_PO_4_; 10 mM Tris·Cl; 8 M urea pH 8). The cells were lysed by 3 cycles of freeze-and-thaw in liquid nitrogen and cold-water bath. Then, the sample was subjected to sonication with 5 s ON and 15 s OFF cycles for 90 s on ice water bath. The cell lysate was cleared by centrifugation for 15 min, 4°C, 10 000 rpm. The supernatant was transferred to a new vial and 2 ml of lysis buffer-washed Ni-resin (Qiagen, Hilden, Germany) was added. The binding was carried out at 4°C for 20 min. The Gli3RD bound resins were collected by centrifugation for 10 min, 4°C, 6 000 rpm. The resin was washed 2 times with 10 ml of washing buffer (50 mM NaH_2_PO_4_; 300 mM NaCl; 20 mM imidazole; pH 8) and collected by centrifugation for 10 min, 4°C, 6 000 rpm. The Gli3RD was eluted with 2 ml of elution buffer (50 mM NaH_2_PO_4_; 300 mM NaCl; pH 4,5) and then further purified with C_18_ reversed-phase chromatography (described in Methods S1) and lyophilized until used. The reversed-phase chromatograms are shown in Figure S1 and Figure S3, the mass spectrum is shown in Figure S2. The final yield of purified Gli3RD was 4 mg per 1 g of biomass, the SDS-PAGE shown in Figure S4.

The soluble Ski was expressed in BL21 cells that have been transformed with pMal plasmid (New England Biolabs, Ipswich, MA). Ski was tagged with MBP and GFP to facilitate expression and purification process. The expression of the recombinant protein was indused with 0.5 mM IPTG for 16 h at 20°C in LB medium. The centrifuged cells were resuspended in 40 ml of ice-cold column buffer (20 mM Tris-HCl; 200 mM NaCl; 1 mM EDTA pH 7.4). Cells were lysed with lysozyme 1 mg/ml for 30 min on ice/water bath, followed by freeze-and-thaw and sonication as for Gli3RD. The cleared lysate was incubated with 1 ml of column buffer washed amylose resin (New England Biolabs). The binding was carried out at 4°C for 20 min. Ski-bound resin was washed 2 times with 10 ml of column buffer and eluted with 1 ml column buffer containing 10 mM maltose. The eluate was kept at 4°C until further use and concentrated to 0.05 mM on Amicon Ultra-0.5 mL Centrifugal Filters (Millipore, Billerica, MA, USA).

### Immunoprecipitation

To test the association of Gli3RD protein and Ski protein we immunoprecipitated one protein and detected whether the other one was co-precipitated. Both proteins were over-expressed as tagged proteins in 293HEK cells. The cells were transfected in 15 cm culture dishes. For transfection the DNA/PEI complex was prepared as follows: 30 µg of Gli3RD or Ski encoding DNA was mixed with 60 µg of PEI per plate in 500 µl of DMEM. After 10 min of incubation 9 ml of DMEM was added to the DNA/PEI mixture and then applied to the culture dish. After 2 h the medium was exchanged for DMEM supplemented with 10% FCS. On the following day, the medium was changed again and after an additional 24 h cells were lysed in 1 ml PBS with 1% Triton X-100 (Sigma–Aldrich). The lysate was cleared by centrifugation at 13 000 rpm for 15 min at 4°C.

For immunoprecipitation of Gli3RD, 5 µl of anti-Gal4 polyclonal antibodies (Santa Cruz Biotechnology, Santa Cruz, CA, USA) or for Ski, 5 µl of anti-GluGlu polyclonal antibodies (Abcam, Cambridge,UK) were incubated with 30 µl Protein G agarose (Amersham Biosciences, Bucks, UK) for 1 h at 4°C. After that 1 ml of cell lysate was added and immunoprecipitation was performed at 4°C overnight and analyzed by Western blot using Gal4 monoclonal antibodies (Santa Cruz Biotechnology), to detect Gli3RD or GluGlu monoclonal antibodies (Abcam, Cambridge,UK) to detect Ski.

### Electrophoretic Mobility Shift Assay

To test the association of Gli3RD with DNA we incubated cell lysate of HEK293 cells expressing Gli3RD and oligonucleotides containing either Gal4 binding site (Gal4BS) or scrambled sequence. The transfected cells were washed with PBS and cells were collected from the 10 mm plate in 0.5 ml of whole cell extraction buffer (20 mM Hepes-KOH, pH 7.9, 400 mM KCl, 1 mM EDTA, 10% (v/v) Glycerol, with freshly added 10 mM DTT, 1 mM PMSF and 1× Complete protease inhibitor cocktail, Roche Diagnostics GmbH, Mannheim, Germany). The cells were lysed in 3 cycles of freezing and thawing in liquid nitrogen and ice bath. The lysate was cleared by 15 min centrifugation, 4°C, 14 000 rpm. The supernatant was aliquoted and kept in −80°C until needed. The untransfected cells used as a negative control were treated likewise.

The two strands of Gal4BS or scrambled oligonucleotides were annealed and labelled with Klenow fragment of DNA polymerase (Bioron, HeidelbergGermany). The labelling reaction was set as follows: 10.5 µl H_2_O, 1.5 µl Reaction buffer (Bioron), 1 µl oligonucleotides (from 4 µM annealed stock), 1 µl [α-^32^P] dCTP (GE Healthcare, UK) and 1 µl Klenow. The reaction was incubated for 30 min at 37°C. Then, 2 µl dCTP (10 mM stock) was added and incubated for an additional 10 min at 37°C. For purification of labelled oligonucleotides from unincorporated label 80 µl Tris-EDTA (10 mM Tris-HCl, pH 7.5; 1 mM EDTA) was added to the probe and the total of 100 µl was run over a NAP-5 Sephadex G25 column (GE Healthcare, London, UK). The column was washed with 400 µL Tris-EDTA. Additional 0.5 ml of Tris-EDTA was added to elute the labelled oligonucleotides.

Band shift binding reactions were assembled by adding 10 µl binding buffer (2x stock: 100 mM HEPES, pH 7.4; 100 mM KCl; 10 mM MgCl_2_; 20 µM ZnSO_4_; 2 mM DTT; 40% glycerol) [2], 1 µl cell lysate, 2 µl labelled Gal4 BS or scrambled oligonucleotides and H_2_O to the total volume of 20 µL. While using Gal4BS oligonucleotides 1 µl of poly-(dI-dC) (1 µg/µL, Sigma) was added to eliminate unspecific binding to DNA. To test the specificity of the binding complex 2 µl (from 4 µM stock) of unlabelled Gal4BS oligonucleotides or unlabelled scrambled oligonucleotides were added, and preincubated for 15 min at room temperature. Then all the band shift binding reactions were incubated at room temperature for 15 min and loaded on 5% nondenaturing polyacrylamide gel in 1x TBE buffer. Before loading, the gel was pre run for 1.5 h, 100 V at 4°C. After loading, the gel was run for 2 h, 200 V at 4°C. and then dried on a Whatman paper (Whatman Ltd, Maidstone, UK) and visualised on a Roentgen film (Agfa HealthCare,Mortsel, Belgium). The gel and the film were assembled in cassette (Kodak, New York, NY USA) and exposed at −70°C for 24 hours.

### Cell culture

HEK293 cells were grown and transfected as previously described [7]. The cells were maintained in Dulbecco's modified Eagle's medium (DMEM) supplemented with 10% Fetal Calf Serum FCS (PAA Laboratories, Pasching, Austria), streptomycin and penicillin (100 units/ml; Invitrogen, Carlsbad, CA). Cells were grown at 37°C and 5.0% CO_2_ in cell culture incubator. One day before transfection the cells were splitted into the required plates.

### Luciferase assays

A DNA plasmid encoding luciferase gene with upstream Gal4 binding sites was used as a reporter. The effector plasmid contained Gli3RD DNA, cloned in frame with Gal4 DNA binding domain (DBD). The plasmids were transfected into mammalian cells. The effector plasmid expressed a fusion protein consisting of Gal4 DBD and Gli3RD. This fusion protein bind to the Gla4 binding sites of the reporter plasmid through the Gal4 DBD and alters the expression of the liciferase gene. We compare the activity of the reporter in the presence and absence of Gla4-Gl3RD. Transfections for luciferase assays were performed in 24-well plates. The Gli3RD WT and mutated constructs were transfected in HEK293 cells. For transfection efficiency control the β-gal construct was used. The amount of reporter plasmid (pMN-Luc) and the effector plasmids (pFA Gal4 fusions expressing RD or RD mutants) used were 300 ng and 100 ng per well, respectivly. For normalization we used 50 ng of pCMV-β-gal. As a transfecting agent we used Polyethyleneimine (PEI; Sigma-Aldrich, St. Louise, USA) 1 µg per well. DNA and PEI were mixed in 50 µl of DMEM. An additional 150 µl of DMEM was added to the DNA/PEI mixture and then applied to the cells. After 2 hours of incubation the medium was exchanged by DMEM with 10% FCS. On the following day the medium was changed again. Cells were harvested after an additional 24 hours of incubation. Firefly luciferase and β-gal assays were performed in Ascent FL fluoroscan with the Luciferase Assay Kit (BioTherma, Dalarö, Sweden) and Galacto-Light Plus System (Applied Biosystems, Foster City, CA). One-Way ANOVA with Dunnett's Muliple Comparisons Test (GraphPad) was used to determine the statistical significance of differential findings between experimental groups.

### CD and NMR analyses of Gli3RD

The in-cell NMR measurements were carried out with cells prepared as described by Serber [37]. The recombinant *E.coli* was grown to OD_600_∼1 in LB medium, then the cells were pelleted by centrifugation (3750 g for 3 min) and transferred to the expression medium (M9) containing ^15^NH_4_Cl and glycerol. After induction with 0.5 mM IPTG for 7 h at 30°C the cells were pelleted (20 min at 800 g), transferred to PBS buffer containing 10% D_2_O and kept on ice until the NMR measurements.

For NMR measurements the lyophilised Gli3RD was resuspended in 50 mM phosphate buffer pH 5.8 or PBS containing 10% D_2_O, to a final concentration of 0.13 mM. NMR spectra were acquired using a Bruker Avance III spectrometer operating at 800 MHz proton resonance frequency. ^1^H-^15^N HSQC spectra were recorded using 79 ms acquisition time in the ^1^H dimension and 51 ms acquisition time in the ^15^N dimension. 4 repetitions were averaged for 256 increments in the indirect dimension. Spectral widths were 8000 Hz for ^1^H and 2500 Hz for the ^15^N dimension. All NMR data processing was performed using TopSpin software (Bruker, Germany).

The CD measurements of DNA binding to Gli3RD were assembled in binding buffer (20 mM HEPES pH 7.9, 80 mM KCl and 4 mM MgCl_2_) by adding equimolar concentrations of lyophilised protein and 21 bp scrambled sequence synthetic oligonucleotides or a plasmid DNA to exclude DNA size requirements. The concentration of the protein was measured spectrophotometrically on ND-1000 (NanoDrop Technologies, Wilmington, DE, USA). CD spectra were obtained with a Jasco J-720 spectropolarimeter (Jasco, Easton, MO) and the temperature was controlled with a PTC-343 temperature controller. A quartz cell with 2 mm optical path was used. The spectral range was 190 – 250 nm with a resolution of 0.2 nm and a bandwidth of 2 nm. A scan speed of 50 nm/min with 2 s response time was employed. The buffer-background spectrum was subtracted.

## Supporting Information

Figure S1 **The UV chromatogram of the sample Gli3RD, 280 nm.** The peak RT 16,52 min was identified as protein with MW 15570.9 Da on the basis of the ESI-MS spectrum deconvulated with the MagTrans software.
(TIF)

Figure S2 **The ESI-MS spectrum of Gli3RD.**
(PDF)

Figure S3 **Overlaid chromatograms of the sample Gli3RD.** Black - UV 280 nm, red - TIC m/z 300 – 2000 Da.
(TIF)

Figure S4 **The SDS-PAGE of Gli3RD. The gel was made to 12% acrylamide, bis-acrylamide 29∶1.**
(JPG)

Methods S1 **HPLC purification.**
(DOC)
