# Supplementary figures and images for: The Transcriptional Repressor Domain of Gli3 Is Intrinsically Disordered
Source: PLoS One. 2013 Oct 17;8(10):e76972. doi: 10.1371/journal.pone.0076972 (PMC3798401; doi:10.1371/journal.pone.0076972)

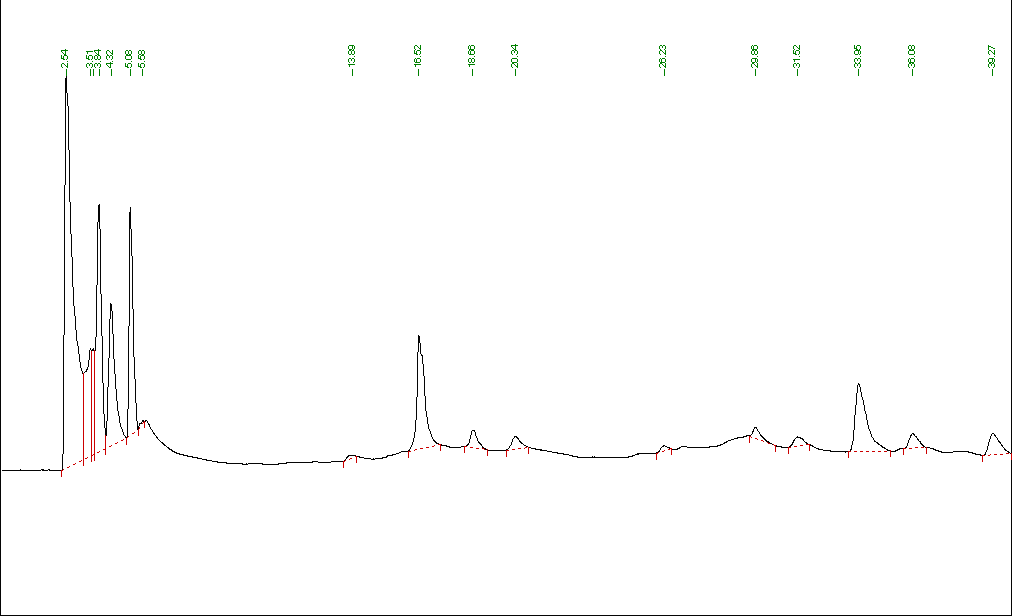

Supplement: Figure S1 — The UV chromatogram of the sample Gli3RD, 280 nm. The peak RT 16,52 min was identified as protein with MW 15570.9 Da on the basis of the ESI-MS spectrum deconvulated with the MagTrans software. (TIF) [file pone.0076972.s001.tif]

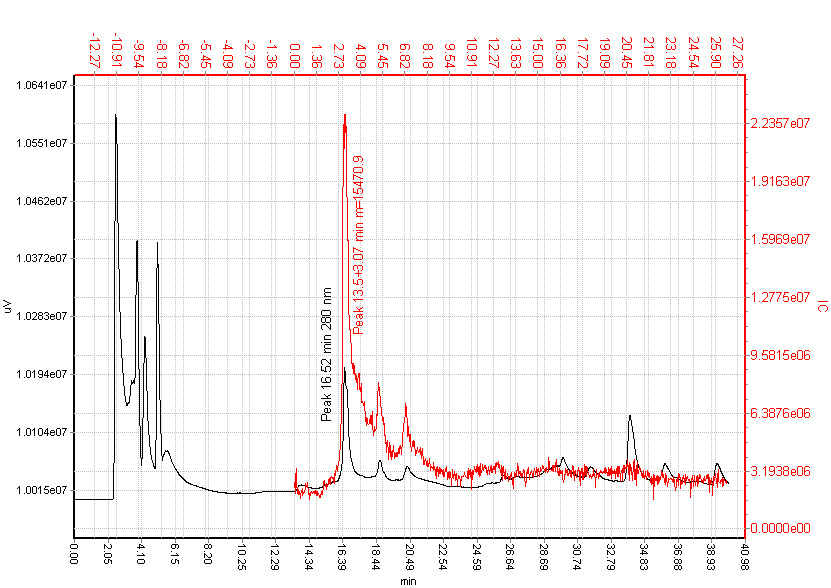

Supplement: Figure S3 — Overlaid chromatograms of the sample Gli3RD. Black - UV 280 nm, red - TIC m/z 300 – 2000 Da. (TIF) [file pone.0076972.s003.tif]

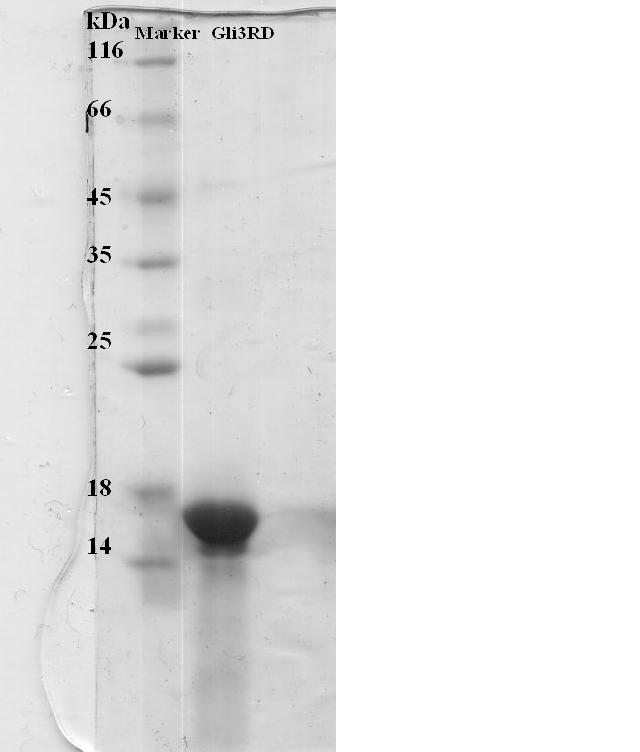

Supplement: Figure S4 — The SDS-PAGE of Gli3RD. The gel was made to 12% acrylamide, bis-acrylamide 29∶1. (JPG) [file pone.0076972.s004.jpg]
