# Supplementary material for: The Transcriptional Repressor Domain of Gli3 Is Intrinsically Disordered
Source: PLoS One. 2013 Oct 17;8(10):e76972. doi: 10.1371/journal.pone.0076972 (PMC3798401; doi:10.1371/journal.pone.0076972)

Magic Transformer v1.0 -- 05/31/12, 11:10

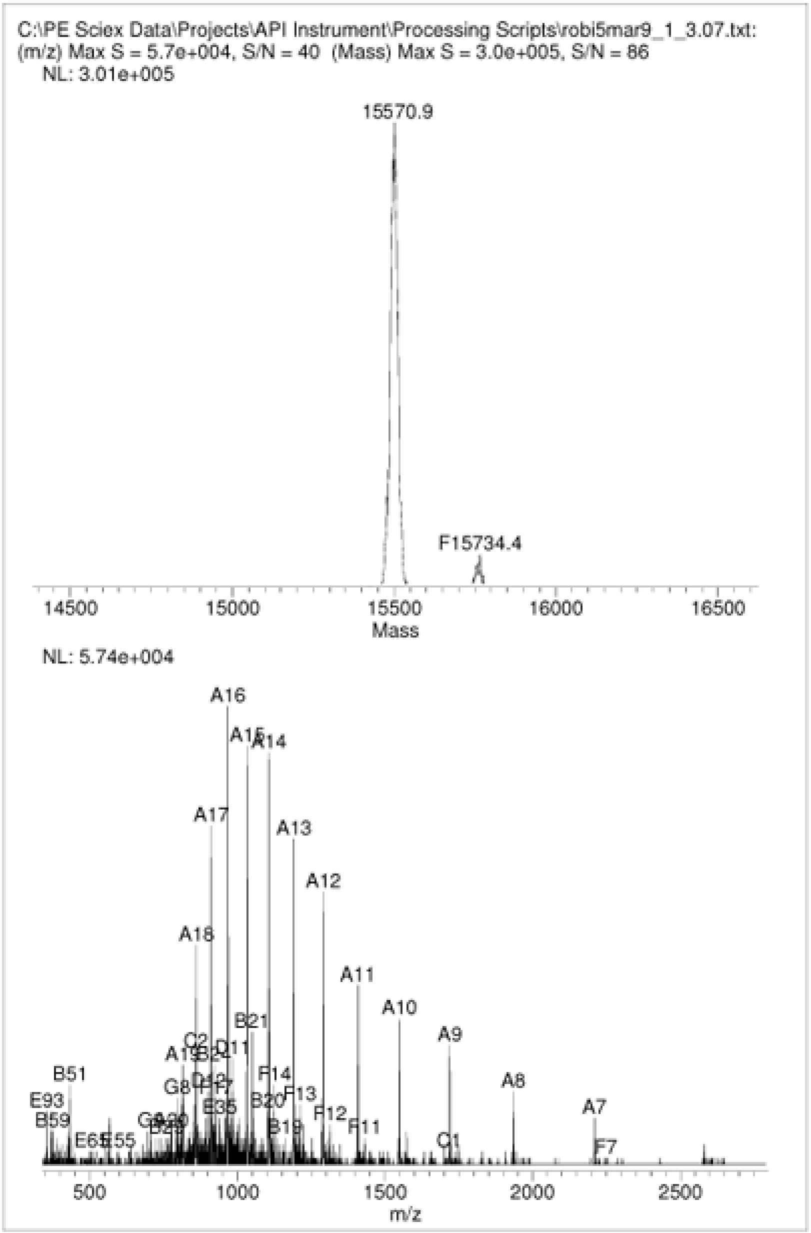

Supplement: Figure S2 — The ESI-MS spectrum of Gli3RD. (PDF) [file pone.0076972.s002.pdf]
