# Supplementary material for: The Transcriptional Repressor Domain of Gli3 Is Intrinsically Disordered
Source: PLoS One. 2013 Oct 17;8(10):e76972. doi: 10.1371/journal.pone.0076972 (PMC3798401; doi:10.1371/journal.pone.0076972)
Supplement: Methods S1 — HPLC purification. (DOC) [file pone.0076972.s005.doc]

Methods S1 HPLC purification

After the affinity chromatography the obtained Gli3RD eluation solution (portions by 1 ml) was passed through the preliminary washed (acetonitrile) and equilibrated (water) SPE cartridge OASIS HLB VAC 60 mg (Waters), followed by 0,5 ml of 10% (v/v) acetonitrile in water and the absorbed matter washed out with 1 mL of the mixture of acetonitrile:water 1:1 (v/v). The eluted solution was concentrated down to ~100 uL in the flow of nitrogen 40°C and injected into the HPLC instrument Series 200 (Perkin Elmer) equipped with diod-array UV/VIS detector and the column Shimpack ODS CLC 15 cm x 6 mm ID (Shimadzu). The mass spectrometer API 150 EX (Sciex) was used as additional detector connected to the outflow from the column through flow splitter Starret No. 460M (Upchurch Scientific) regulated to the flow 100 uL/min into the MS. Initial HPLC eluent (A) was 10% of acetonitrile and 0.1% trifluoroacetic acid in water (solvent A) followed by linear gradient of the 0.1% TFA in acetonitrile (B) up to 90% in 30 min (all concentrations v/v), flow rate 1 mL/min, column temperature 35°. Gli3RD peak on the chromatogram (Figure 3) was identified on the basis of the mass spectrum (Figure 2) and collected by hand acccording to the UV signal at 280 nm, avoiding collection of impurities closely following to the main peak (red TIC track on the figure 3). All fractions of pure Gli3RD were joined concentrated up to removal of acetonitrile and lyophilized until used.

TIC = Total Ion Count
